# The impact of T1 versus EPI spatial normalization templates for fMRI data analyses

**DOI:** 10.1002/hbm.23737

**Published:** 2017-07-26

**Authors:** Vince D. Calhoun, Tor D. Wager, Anjali Krishnan, Keri S. Rosch, Karen E. Seymour, Mary Beth Nebel, Stewart H. Mostofsky, Prashanth Nyalakanai, Kent Kiehl

**Affiliations:** ^1^ The Mind Research Network & LBERI Albuquerque New Mexico; ^2^ Department of ECE University of New Mexico Albuquerque New Mexico; ^3^ Department of Psychology University of New Mexico Albuquerque New Mexico; ^4^ University of Colorado at Boulder Boulder Colorado; ^5^ Center for Neurodevelopmental and Imaging Research, Kennedy Krieger Institute Baltimore Maryland; ^6^ Department of Psychiatry and Behavioral Sciences Johns Hopkins University School of Medicine Baltimore Maryland; ^7^ Department of Neurology Johns Hopkins University School of Medicine Baltimore Maryland

**Keywords:** spatial normalization, echo planar image, fMRI, coregistration

## Abstract

Spatial normalization of brains to a standardized space is a widely used approach for group studies in functional magnetic resonance imaging (fMRI) data. Commonly used template‐based approaches are complicated by signal dropout and distortions in echo planar imaging (EPI) data. The most widely used software packages implement two common template‐based strategies: (1) affine transformation of the EPI data to an EPI template followed by nonlinear registration to an EPI template (EPInorm) and (2) affine transformation of the EPI data to the anatomic image for a given subject, followed by nonlinear registration of the anatomic data to an anatomic template, which produces a transformation that is applied to the EPI data (T1norm). EPI distortion correction can be used to adjust for geometric distortion of EPI relative to the T1 images. However, in practice, this EPI distortion correction step is often skipped. We compare these template‐based strategies empirically in four large datasets. We find that the EPInorm approach consistently shows reduced variability across subjects, especially in the case when distortion correction is not applied. EPInorm also shows lower estimates for coregistration distances among subjects (i.e., within‐dataset similarity is higher). Finally, the EPInorm approach shows higher *T* values in a task‐based dataset. Thus, the EPInorm approach appears to amplify the power of the sample compared to the T1norm approach when not using distortion correction (i.e., the EPInorm boosts the effective sample size by 12–25%). In sum, these results argue for the use of EPInorm over the T1norm when no distortion correction is used. *Hum Brain Mapp 38:5331–5342, 2017*. © **2017 The Authors Human Brain Mapping Published by Wiley Periodicals, Inc.**

## INTRODUCTION

Spatial normalization of fMRI data has been broadly implemented in a variety of widely used software packages including AFNI (http://afni.nimh.nih.gov/afni), SPM [Friston, 2005], and FSL (http://www.fmrib.ox.ac.uk). One of the challenges of working with the echo planar imaging (EPI) scans is that they suffer from geometric distortion and signal dropout. Two main template‐based approaches have been utilized to spatially normalize EPI data into standard space (e.g., Montreal Neuroimaging Institute (MNI) standard space) [Collins et al., 1994; Mazziotta et al., 2001, 1995; Talairach and Tournoux, 1988; Tzourio‐Mazoyer et al., 2002].

The first approach, called EPInorm, involves an affine transform followed by a nonlinear registration of the EPI image to an EPI template in standard space (we use MNI from this point forward though the approach can apply to any template) [Fox, 1995; Friston et al., 1995a, 1995b, 1999; Gaser et al., 1999; Huntenberg et al., 2014; Klein et al., 2009; Woods et al., 1998]. An advantage of this approach is that it directly addresses the nonlinearities the EPI image exhibits, but a potential drawback is that it can suffer from over correction (e.g., pulling of unrelated brain regions to fill regions of signal dropout).

**Figure 1:**
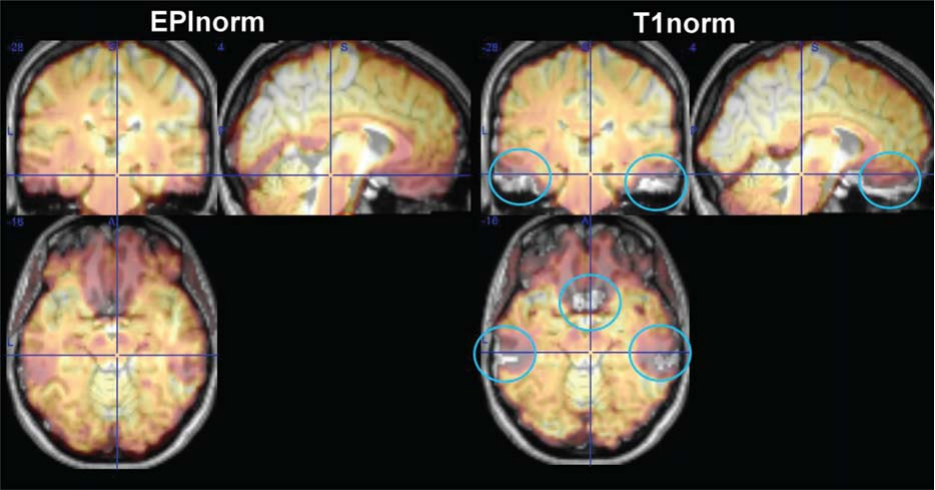
Side‐by‐side comparison of EPInorm and T1norm approaches for a single subject transparently overlaid on the T1 image from that subject. The T1norm process is unable to compensate for distortions throughout the brain, which are not present in the T1 scan (blue circles). [Color figure can be viewed at http://wileyonlinelibrary.com]

The second approach, called T1norm, includes the estimation of an affine transform mapping between the EPI image to the T1 image for that individual followed by a nonlinear warp between the T1 and a T1 MNI template. These warp parameters are then applied to the coregistered EPI image resulting in MNI normalized EPI data [Fox, 1995; Friston et al., 1995; Klein et al., 2009; Tzourio‐Mazoyer et al., 2002]. An advantage of this approach is that it typically relies on an image with higher spatial resolution to estimate the nonlinear warp to MNI space. However, a potential drawback of this approach is that it does not account for the geometric distortions (which can be substantial) that impact the EPI data, but not the T1 data, as it assumes the affine transform can correct for any differences between the EPI and T1 data from the same subject (Fig. 1).

Both the above approaches can be applied to raw EPI data or on distortion‐corrected EPI data. Geometric distortion can be corrected to a degree using field maps [Jezzard and Balaban, 1995] or interpolation between images calculated using multiple phase encode directions [Holland et al., 2010]. In this case, the T1norm approach makes more sense as the nonlinear distortion correction provides a physics‐based correction of the nonlinearities between the T1 and the EPI data. Despite this, presumably because collection of and/or use of the field map adds additional complexity to the data collection and post processing, the vast majority of fMRI studies published use the T1norm approach and do not report using geometric distortion correction. When distortion correction is not performed, the spatial resolution advantages of the T1norm approach are likely outweighed by the disadvantages caused by distortion discrepancies between individual subjects' EPI and T1 data; poor coregistration between the EPI and T1 data may result in EPI data that are not well aligned across subjects. While several studies have compared nonlinear warping procedures commonly used within the T1norm framework [Ardekani et al., 2004, 2005; Crinion et al., 2007; Ghosh et al., 2010; Tahmasebi et al., 2009], to our knowledge, no study has systematically compared the EPInorm and T1norm approaches.

The goal of this article is to evaluate EPI spatial normalization schemes in group studies using either the EPInorm or the T1norm approaches. To do this, we evaluate four different datasets using several metrics. One outcome metric is the variability of the coregistration across subjects. This measure estimates the degree of similarity between the spatially normalized images from different individuals. A second metric is the coregistration between spatially normalized images across individuals. Finally, using a go/no‐go task, we evaluate the impact of these various approaches on the resulting *T* values, as increasing the anatomic accuracy of spatial normalization has been shown to increase sensitivity for detecting task‐related activation as well as the replicability of activation maps [Ardekani et al., 2005]. We focus primarily on the template‐based nonlinear spatial normalization within the SPM12 pipeline, but results are expected to apply to any T1norm approach. For comparison, we also include preprocessed data from the Autism Brain Imaging Data Exchange (ABIDE [Craddock, 2013; Martino et al., 2013]) which differed in the way that EPI and T1 data were aligned and in the way that T1 data were transformed to standard space (http://preprocessed-connectomes-project.org/abide/). The nonlinear boundary based registration (BBR) approach in FSL 5.0 was used to align EPI and T1 data, and linear and nonlinear approaches in the advanced normalization tools (ANTs) software package [Avants et al., 2008] were used to transform T1 data to standard space. BBR includes field inhomogeneity correction and aligns the EPI to the T1 by maximizing the intensity gradient across tissue boundaries [Greve and Fischl, 2009]. ANTs has been demonstrably more accurate in published studies comparing T1 normalization strategies than standard voxelwise normalization [Avants and Gee, 2004; Avants et al., 2011].

## METHODS

We focus on a comparison of EPInorm and T1norm for four different datasets, processed a variety of different ways. The data are briefly described in Table 1 and include an 81‐subject multiband dataset collected during a go/no‐go task at the Mind Research Network and analyzed without (Experiment 1a) and with (Experiment 1b) distortion correction, a 30‐subject pain dataset previously published using a T1norm approach [Krishnan et al., 2016] (Experiment 2), a pediatric study including 112 typically developing 8–12‐year‐old children (Experiment 3), and the ABIDE [Craddock, 2013; Martino et al., 2013] data, which is an 1100‐subject multisite autism dataset (age range 5–64) processed using an FSL T1norm pipeline (Experiment 4).

**Table 1:** Summary of datasets

	# Participants, data type	Distortion corrected	EPInorm	T1norm	Output
Ex 1a: Multiband	81 go‐nogo task	Yes	SPM^a^	SPM^a^	Subjectwise variance map, T‐map
Ex 1b: Multiband	81 go‐nogo task	No	SPM^a^	SPM^a^	Subjectwise variance map, T‐map
Ex 2: CU Boulder	30, pain task	No	SPM^a^	SPM^a^	Subjectwise variance map
Ex 3: KKI	112, rest fMRI	No	SPM^a^	SPM^a^	Coregistration subject‐to‐template similarity
Ex 4: ABIDE multisite	1100, rest fMRI	No	SPM^a^	FSL^b^	Subjectwise variance map

a SPM12, 4 × 5 × 4 basis set, interpolated to 3 × 3 × 3 mm voxels.

b FSL used the boundary‐based registration approach described in the ABIDE preprocessing initiative [Friston et al., 1999].

**Figure 2:**
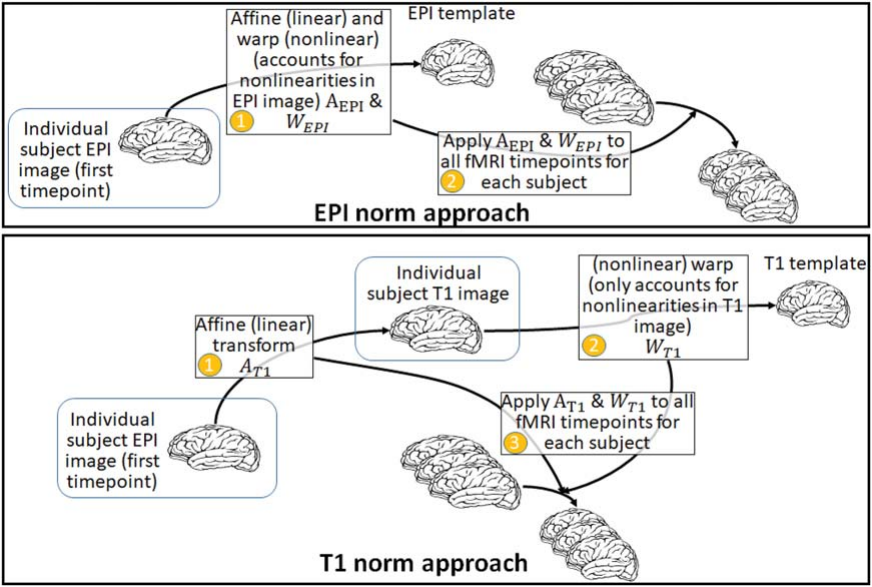
Schematic of the EPInorm and T1norm approaches. [Color figure can be viewed at http://wileyonlinelibrary.com]

The data are slice‐time corrected, motion corrected, and then spatially normalized in SPM using two approaches. For the EPInorm approach, we spatially normalize using the EPI MNI template as the target using a 4 × 5 × 4 basis set to mitigate overfitting. This approach involves an initial affine transform followed by a nonlinear warp, resulting in a nonlinear matching of the EPI image to the template. The T1norm approach involves an affine transform from the EPI image to the T1 image (or vice versa) from the same subject. In the case of the ABIDE dataset, the EPI data are registered to the T1 image with a linear transformation, followed by a white‐matter boundary based transformation using FMRIB's Linear Image Registration Tool (FLIRT) [Jenkinson and Smith, 2001] and then the prior white‐matter tissue segmentation from FMRIB's Automated Segmentation Tool (FAST) [Zhang et al., 2001]. Next, the T1 image is warped to the template using the SPM T1 MNI template via the unified segmentation approach [Ashburner and Friston, 2005], or in the case of the ABIDE dataset, linear and nonlinear transformations to MNI are performed using ANTs. The resulting warp parameters are applied to the EPI image, producing functional images in standard MNI space. See Figure 2 for a schematic demonstrating the two approaches. Additionally, for the multiband dataset, we collected a distortion correction set, which consists of two images acquired with different phase encode directions. These are used to distortion correct the EPI data using the FSL topup program [Andersson et al., 2003] and compared with the images that were not distortion corrected.

### Metrics

We use several straightforward metrics for comparison in this study. The first metric used (Experiments 1, 2, and 4) was based on an evaluation of the voxelwise variability of the coregistered images across subjects. Within each dataset/experiment, we extracted the first timepoint of each subject's fMRI data, divided by the in‐brain mean for that subject and multiplied by 100. Next, we compute the voxelwise standard deviation among subjects. This provides a measure of the variability of the image; if a given voxel is always inside the brain at a voxel with a value of 100, the standard deviation will be zero. If a given voxel is on the edge and varying constantly between being “in” and “out” of the brain, this voxel will tend to have a high standard deviation. In an ideal case, all the datasets would be perfect matches to the MNI atlas and the standard deviation would be very low. In reality, we expect more variability at the edges of the brain, which largely reflects mismatch in the alignment of brains across individuals.

A second metric we use (Experiment 3) was the mean subject‐to‐subject displacement. That is, using the SPM image alignment algorithm, we compute the average displacement among each subject relative to a random subject in the dataset. Specifically, the first smoothed volume of the reference subject was concatenated with the first smoothed functional volume of every other subject along the fourth dimension to create a single image file with 112 frames. The rigid body realignment parameters were then estimated on this 4D file created for each normalization method. Next, we calculated the sum of the absolute value of the six realignment estimates for each frame (subject) relative to the first frame (reference subject) after converting the three rotational displacements from degrees to millimeters by assuming a 50‐mm radius from the cortex to the center of the head.

A third metric used was the task‐activation effect size. For Experiments 1 and 2, we computed a GLM model fit using SPM and compared the resulting *T* values for the main effect of interest (false alarms versus hits) in a go/no‐go task. We then tested for differences in the within subject mean of the *T* value above a given threshold (e.g., *T* = 4). In addition, under certain assumptions, we can calculate the number of subjects needed to match the T‐values. That is, if $T_{1}=\mu_{1}/(\frac{\sigma_{1}}{\sqrt{N_{1}-1}})$ and $T_{2}=\mu_{2}/(\frac{\sigma_{2}}{\sqrt{N_{2}-1}})$, then given a calculated value for $T_{1}$and $T_{2}$ and assuming the mean and standard deviations are the same (i.e., $\mu_{1}=\mu_{2}$ and $\sigma_{1}=\sigma_{2}$, we can calculate the number of subjects needed to make the *T* values equal, i.e., $\frac{T_{1}}{T_{2}}=\sqrt{N_{1}-1}/\sqrt{N_{2}-1}$ or $N_{eff}={\left(\frac{T_{1}}{T_{2}}\right)}^{2}\left(N_{ref}-1\right)+1$. Or more specifically, if $T_{1}>T_{2}$ this will enable us to calculate how many more subjects we would have needed to collect ( N_eff) to find equivalent T‐values to the reference case ( $N_{ref}$).

## RESULTS

### Voxel Variability as a Measure of Intersubject Alignment

**Figure 3:**
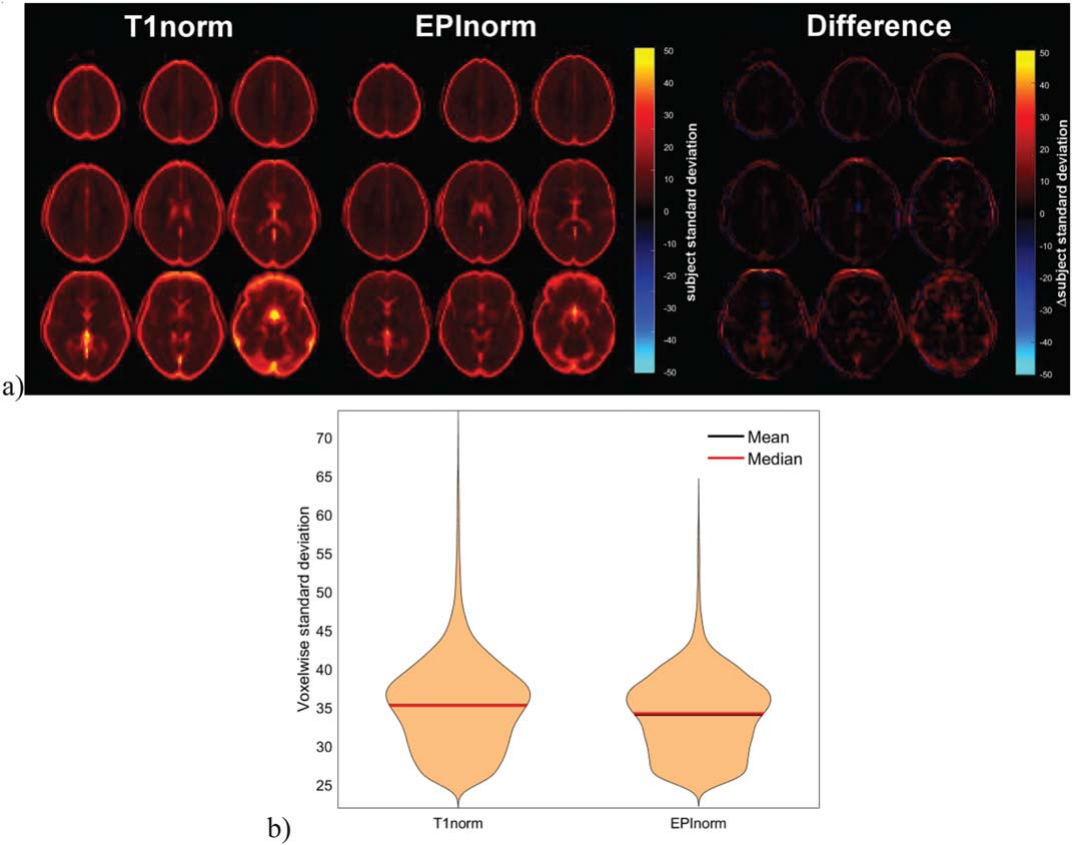
T1norm versus EPInorm in Experiment 1a: (a, left) T1 norm voxelwise subject standard deviation, (a, middle) EPInorm voxelwise subject standard deviation, (a, right) difference (T1norm–EPInorm). (b) Violin plot of the voxels showing a subject standard deviation of over 25, T1norm shows a higher whole brain mean. [Color figure can be viewed at http://wileyonlinelibrary.com]

The voxelwise standard deviation metric showed that intersubject alignment was worse (indicated by significantly higher voxelwise standard deviations) using the T1norm approach than using the EPInorm approach. This was true for all datasets regardless of whether (a) distortion correction was performed; (b) functional data were collected using a standard EPI pulse sequence or a multiband pulse sequence; or (c) whether we implemented the T1norm approach ourselves or relied on publicly available preprocessed data using this approach. For Experiment 1a (go/no‐go fMRI data processed without distortion correction), the mean + standard deviation was lower for the EPInorm approach (EPInorm: 5.9 ± 8.0, T1norm: 7.4 ± 9.7). Figure 3a shows the standard deviation images for T1norm and EPInorm without distortion correction and the difference. Figure 3b shows the violin plots of the mean across the image and indicates that the EPInorm mean is lower than the T1norm mean. In both cases, it is clear that the T1norm approach shows more voxels with larger subject standard deviation values.

**Figure 4:**
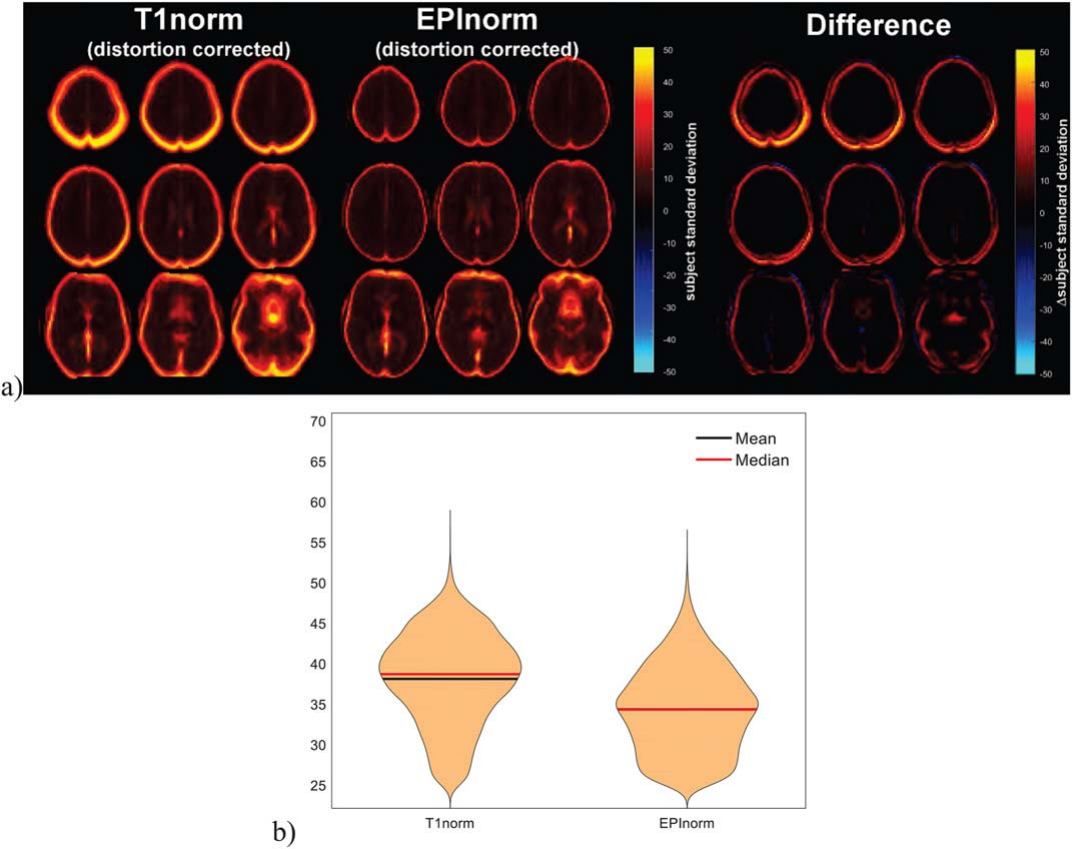
T1norm versus EPInorm in Experiment 1b: (a, left) T1 norm voxelwise subject standard deviation, (a, middle) EPInorm voxelwise subject standard deviation, (a, right) difference (T1norm–EPInorm). (b) Violin plot of the voxels showing a subject standard deviation of over 25, T1norm shows a higher whole brain mean. [Color figure can be viewed at http://wileyonlinelibrary.com]

Voxelwise standard deviations for Experiment 1b (go/no‐go fMRI data processed with distortion correction) are shown in Figure 4a. As was the case for Experiment 1a, more voxels had larger standard deviation values across subjects for the T1norm approach than for the EPInorm approach. Comparing voxelwise standard deviation values across Experiments 1a and 1b, we observed that the T1norm approach with distortion correction showed more subjectwise variability than without distortion correction. This may be due to the fact that the EPI image is collected with a single phase encode direction rather than with both; as such, the distortions (and signal dropout) are somewhat biased to a specific scenario (and not well corrected by the T1norm approach). We thus rank the four processing scenarios for the go/no‐go fMRI data in terms of the whole brain mean and standard deviation for voxel variability in the following order from worst to best: T1norm (with distortion correction): 11.0 ± 13.4, T1norm (without distortion correction): 8.5 ± 11.1, EPInorm (without distortion correction): 8.0 ± 10.5, and EPInorm (with distortion correction): 7.5 ± 10.3.

**Figure 5:**
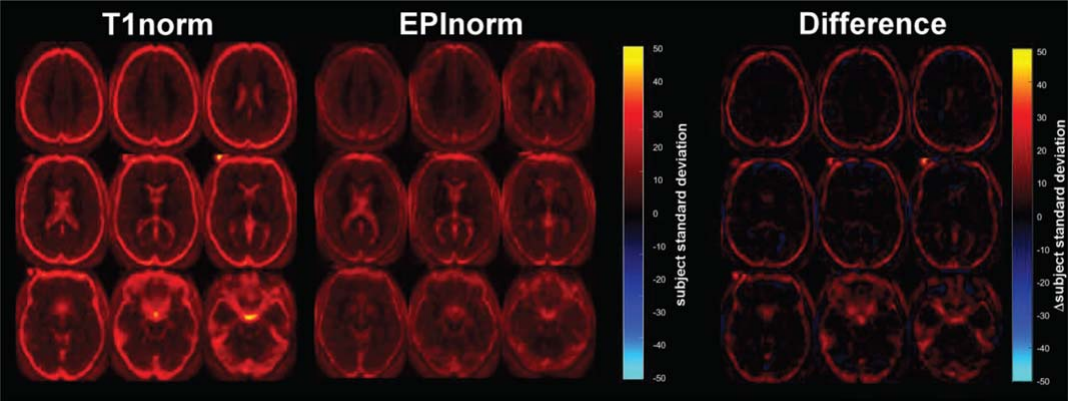
T1norm versus EPInorm in UC Boulder dataset: (left) T1 norm voxelwise subject standard deviation, (middle) EPInorm voxelwise subject standard deviation, (right) difference (T1norm–EPInorm). [Color figure can be viewed at http://wileyonlinelibrary.com]

To ensure that our results for Experiments 1a and 1b were not simply due to our greater familiarity with the EPInorm approach, we additionally evaluated spatial variability across subjects following normalization using 30 subjects from a published dataset that was carefully normalized using the T1norm approach using a manually curated SPM pipeline from Krishnan et al. [2016] (Experiment 2). We reanalyzed these data using the EPInorm approach and computed the voxelwise subject standard deviation for comparison purposes. As before, the T1norm resulted in a higher mean and standard deviation (10.2 ± 8.8) than the EPInorm approach (7.0 ± 5.0). Results are shown in Figure 5. As before, the higher voxelwise subject standard deviation is visible for the individual images as well as for the difference image. In addition, we also reprocessed the data using the same pipeline as Experiment 1 and results were consistent with increased variability for the T1norm approach (results not shown).

### Intersubject Realignment Estimates were Lower for EPInorm Than for T1norm

**Figure 6:**
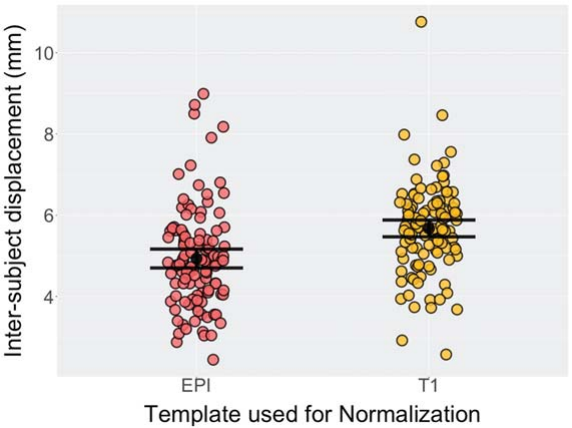
Alignment of first image for each participant relative to a random KKI subject. The EPInorm approach showed significantly more similarity (*P* < 0.05) among subjects in alignment relative to the T1norm approach (Wilcoxon signed‐rank test, *V* = 631, *P* = $1.59\times 10^{-13}$). [Color figure can be viewed at http://wileyonlinelibrary.com]

Experiment 3, a dataset from the Kennedy Krieger Institute, consisted of 112 resting state scans all from typically developing 8–12‐year‐old children. We processed these data using both the T1norm and EPInorm approaches and then assessed between‐subject alignment for each method using the first image from each participant as described in the methods and a random subject from the set as a reference. We compared this measure of intersubject displacement across normalization approaches. Results showing the intersubject image displacement relative to the random subject used as a reference are presented in Figure 6. We observed significantly smaller displacements (indicative of better alignment) between subjects using the EPInorm approach relative to the T1norm approach (Wilcoxon signed‐rank test, *V* = 631, *P* = $1.59\times 10^{-13}$), which is consistent with our voxelwise standard deviation findings.

**Figure 7:**
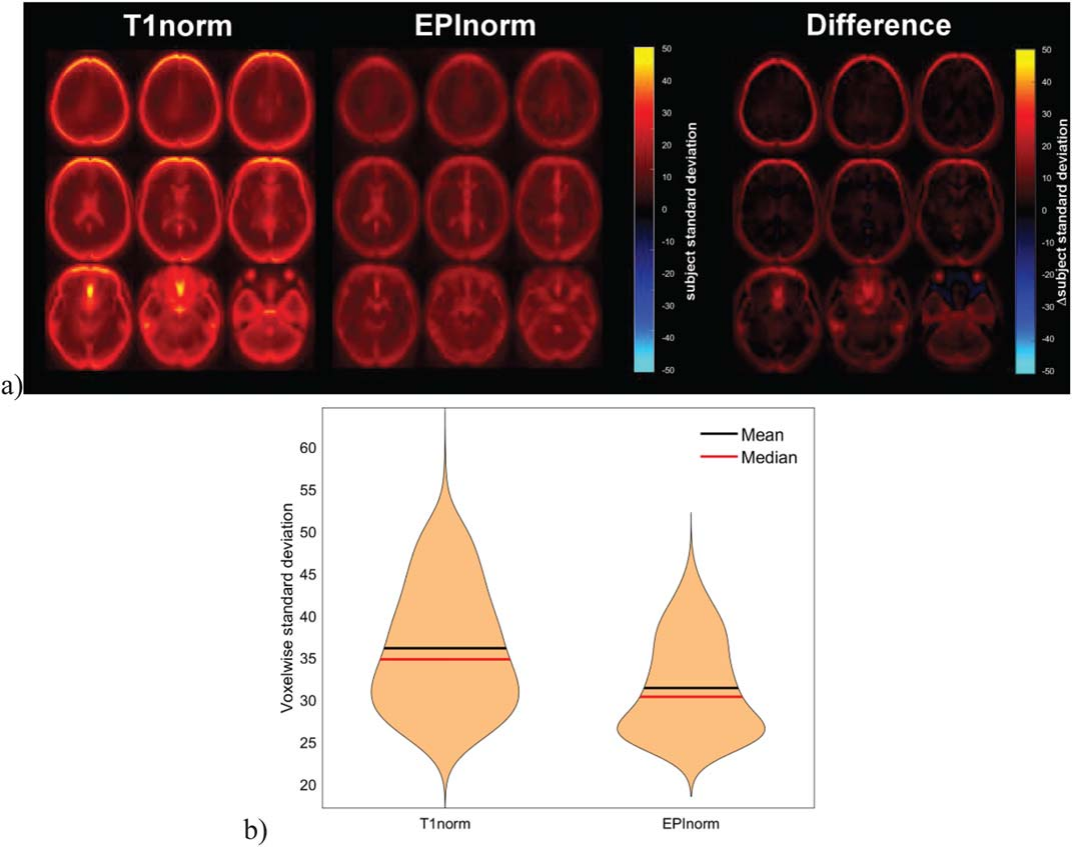
T1norm versus EPInorm in ABIDE dataset: (a, left) T1 norm voxelwise subject standard deviation, (a, middle) EPInorm voxelwise subject standard deviation, (a, right) difference (T1norm–EPInorm). (b) Violin plot of the voxels showing a subject standard deviation of over 25, T1norm is clearly much higher. [Color figure can be viewed at http://wileyonlinelibrary.com]

Experiment 4, the final dataset, ABIDE, consisted of 1100 participants combined via a grass roots multisite consortium. The data were collected in separate studies, rather than harmonized in a coordinated manner. As such, this study has considerably more variability across subjects in parameters, scanner types, and other measures. As part of the ABIDE effort, a preprocessed dataset was released publicly which used FSL's boundary‐based registration via a T1norm approach [Craddock, 2013]. For comparison, we computed the SPM‐based EPInorm approach, and as before, we computed the voxelwise subject standard deviation. Figure 7 again shows the voxelwise subject standard deviation across the brain. As before, the T1norm resulted in higher standard deviations throughout the brain, but especially on the edges of the brain where we expect the highest values. The mean and standard deviation of voxelwise variability for the T1norm and EPInorm approaches were 13.6 ± 9.8 and 9.1 ± 5.7, respectively. A violin plot of the voxel values as well as a scatter plot of T1norm versus EPInorm in Figure 7 shows an even greater difference in variability for both T1norm and EPInorm than for the other datasets evaluated (likely due to the fact that ABIDE is a multisite dataset). We also see the same pattern in which the EPInorm exhibits less variability than the T1norm data in terms of voxelwise subject standard deviation. We also reprocessed the ABIDE data using the same T1norm pipeline as in Experiment 1. Results were consistent in showing greater subjectwise variability using the T1norm compared to the EPInorm approach regardless of whether FSL's boundary‐based registration was used to align each subject's EPI and T1 data followed by nonlinear warping of the T1 data to MNI space or whether SPM's coregistration algorithm was used to align each subject's EPI and T1 data followed by nonlinear warping of the T1 data to MNI space using SPM's unified segmentation normalization procedure (results not shown).

### Assessing the Impact of Intersubject Alignment Strategy on Group‐Level Inferences

**Figure 8:**
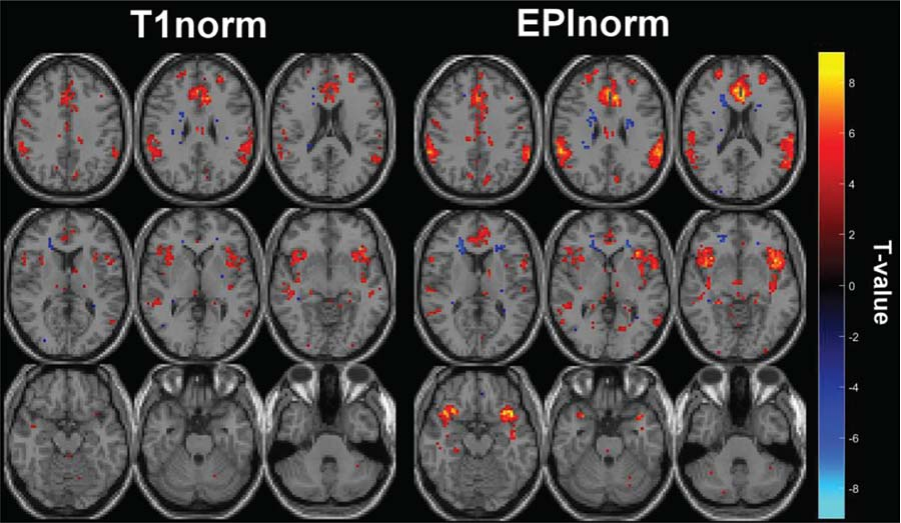
*T* values corresponding to false alarms versus hits for the go/no‐go task without distortion correction for (a) T1norm and (b) EPInorm. [Color figure can be viewed at http://wileyonlinelibrary.com]

To assess the practical impact of variability in intersubject alignment on our ability to draw inferences at the group level, we also analyzed the go/no‐go task data (Experiments 1a and 1b) in SPM. To compare methods, we looked at distributions of *T* values for the false‐alarm‐versus‐hit contrast and calculated effective sample sizes. Figure 8 shows maps of the false‐alarm‐versus‐hit contrast generated from both EPInorm and T1norm in Experiment 1a (without distortion correction; thresholded at *T* > 4.0). It is clear from the figure that the EPInorm approach results in higher *T* values associated with task activity. The maximum (minimum) *T* value for EPInorm is 9.68 (−7.26) and for T1norm is 8.31 (−5.77). If we calculate the average *T* value above 4.0 (to ensure we are comparing effect sizes only in voxels that were strongly task‐related) and test the difference between the two approaches, we find they are significantly different at *P* < 0.05 (*P =* 4.3 × 10^−20^). In addition, as described in the methods, we can calculate the number of subjects needed to obtain a *T* value for the T1norm approach that is equal to that for the EPInorm approach using this mean *T* value. We do this for the single slice in the top right corner of the figure that shows expected activation patterns for the go/no‐go task, and also for the more general whole brain case. This suggests, for the no distortion correction case, an effective sample size of $N_{eff}={\left(\frac{T_{EPInorm}=5.7313}{T_{T1norm}=5.1105}\right)}^{2}80\;+\;1\;=\;102,$ using the EPInorm approach relative to the original $N_{ref}=81$ for the single slice case. Put another way, and subject to the assumptions mentioned earlier, if we take the T1norm mean *T* value as a reference, the EPInorm has amplified the effective N by almost 25%. In the whole brain case, we find $N_{eff}={\left(\frac{T_{EPInorm}=5.4891}{T_{T1norm}=5.1756}\right)}^{2}80\;+\;1\;=\;91$, that is, a 12% “boost” to the effective sample size relative to the T1norm approach. Note that these results are not particularly dependent on the specific *T* value used. We evaluated the change in effective sample size for *T* values between 2 and 5, and in all cases, we observed a higher effective N for the EPInorm approach relative to the T1norm approach without distortion correction. The change in the distribution of voxel values between the two approaches can be observed by comparing the T1norm and EPInorm voxel values show in Figure 8. The averaging approach was used to ensure the effect was consistent across multiple task‐related voxel values rather than selecting only the maximum voxel value (which, if used, showed an even larger benefit of the EPInorm approach compared to the T1norm approach).

**Figure 9:**
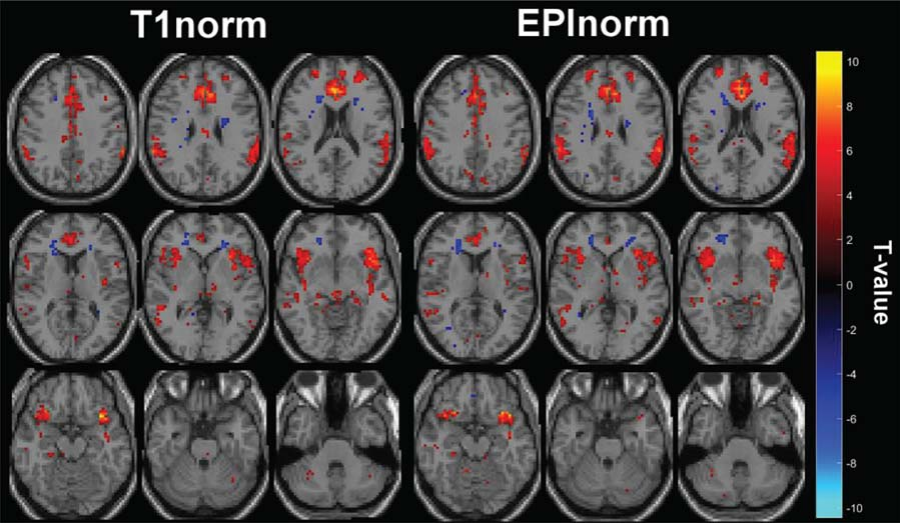
*T* values corresponding to false alarms versus hits for the go/no‐go task with distortion correction for (a) T1norm and (b) EPInorm. [Color figure can be viewed at http://wileyonlinelibrary.com]

Figure 9 shows the maps of the false‐alarm‐versus‐hit contrast generated from both EPInorm and T1norm in Experiment 1b (with distortion correction; thresholded at *T* > 4.0), and qualitatively, the maps for EPInorm and T1norm are much more similar when distortion correction is performed compared to the case without distortion correction in Figure 8. The maximum (minimum) *T* value for EPInorm is 9.73 (−8.18) and for T1norm is 10.45 (−6.30). As before, we calculated the average *T* value above 4.0 and tested the difference between the two approaches with distortion correction but found that this difference was not significant *P* > 0.15. To compare effective sample sizes with distortion correction, we used the T1norm data as the reference because the *T* values were slightly, although not significantly, higher than those from EPInorm with distortion correction. When distortion correction is performed, we calculated the effective number of subjects needed for the EPInorm approach relative to the T1norm approach to be $N_{eff}={\left(\frac{T_{T1norm}=5.4807}{T_{EPInorm}=5.4238}\right)}^{2}80\;+\;1\;=\;83$, relative to the original $N_{ref}=81$ for the single slice case. This represents a 3% boost in the effective sample size. In the whole brain case, we find $N_{eff}={\left(\frac{T_{T1norm}=5.1177}{T_{EPInorm}=5.1384}\right)}^{2}80\;+\;1\;=\;83$, again a 3% increase to the effective sample size relative to the EPInorm approach. Interestingly, the average *T* values for the EPInorm approach without distortion correction were higher than the *T* values for EPInorm with distortion correction (though the maximum *T* value was higher for the EPInorm approach with distortion correction).

### EPInorm Produced Larger *T* Values Than T1Norm Without Distortion Correction

**Figure 10:**
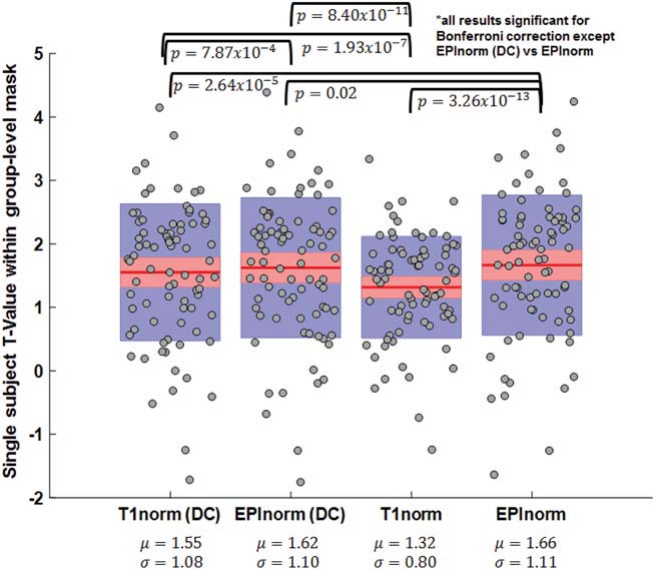
Single‐subject *T* values for the four cases (T1norm, EPInorm, T1norm with distortion correction, and EPInorm with distortion correction): EPInorm shows the highest mean T‐values and T1norm without distortion correction is significantly lower than the other three approaches. [Color figure can be viewed at http://wileyonlinelibrary.com]

Figure 10 shows the *T* values extracted from single subjects. To do this, a mask was created including all voxels for which the group‐level *T* values were larger than 4 for all of the four cases (T1norm, EPInorm, T1norm with distortion correction, and EPInorm with distortion correction). We then calculated the mean of the *T* values within the group mask for each subject. Paired *t* tests showed significant differences among almost all the cases, though, the most significant difference was the T1norm (without distortion correction) compared to the other three cases. The EPInorm approach without distortion correction had a significantly higher mean than both with distortion correction cases for this analysis, though the overall means are relatively close. Reported *P* values (Figure 10) are uncorrected; if we Bonferroni correct for the 6 comparisons, all the results are still significant at *P* < 0.05 with the exception of the comparison between the EPInorm without distortion correction and EPInorm with distortion correction.

## DISCUSSION

Given the large number of studies using either the T1norm or the EPInorm approaches, we compared these two approaches using several straightforward metrics on multiple datasets. Results indicated that the T1norm approach consistently shows higher variability across subjects than does the EPInorm approach. In addition, the inter‐subject realignment estimates were lower for data processed using the EPInorm approach, suggesting more similarity among subjects in alignment relative to the T1norm approach. Finally, the group *T* values generated using go/no‐go data processed using the EPInorm approach were higher than those generated using the T1norm approach. Comparing T‐scores resulting from the two normalization methods suggests that the EPInorm approach effectively amplifies the sample size by between 12% and 25%. The results also suggest that distortion correction substantially improves the T1norm approach, but has less of an impact on the EPInorm approach (which is already doing a type of distortion correction).

Our results also suggest EPInorm without distortion correction provides results as good as or in some cases better than EPInorm or T1norm with distortion correction. This is consistent with previous work which has directly used high resolution EPI data to develop templates and found increased activation as a result [Grabner et al., 2014]. In our case, we show similar enhancement in activation even for standard EPI acquisitions with little T1 contrast. Our results are also consistent with Huang et al. who showed that using a study specific EPI template resulted in greater *t* values and activated voxels within a predefined region of interest [Huang et al., 2010].

A reliance on distortion correction pulse sequences, while attractive as it offers a physics‐based approach for reducing distortions [Jezzard and Balaban, 1995; Holland et al., 2010], suffers from some limitations as well. Studies often collect these data, but in our experience, many do not use them, likely because it adds additional steps to the processing pipeline and also can be further complicated by motion within the run. And, though many studies currently collect distortion correction sequences and share data that has been corrected [Van Essen et al., 2012], there are a huge number of legacy studies which are being shared but did not collect such sequences [Eickhoff et al., 2016]. In addition, despite the relatively simple and short acquisition, many prospective studies still do not collect distortion sequences.

Distortion correction strategies also typically assume there is no motion between the distortion correction sequence and the fMRI acquisition. This can be particularly problematic when participants move their head and no additional distortion correction is collected in this new position. Furthermore, because distortions are different depending on which phase encode direction is used, and most fMRI scans collect only a single phase encode direction, there are limits to what can be corrected. Signal dropout is of course another issue that disproportionally affects EPI data relative to T1 data [Deichmann et al., 2003; Wastling and Barker, 2015]. It would be interesting to design a study to isolate the impact of the geometric distortion and signal dropout properties that the EPI data experience, and to study how these image properties propagate error through the T1norm and EPInorm pipelines. In our opinion, the signal dropout is likely having the largest impact on the results, introducing error in the coregistration between the T1 and EPI data that is then propagated when the transformation to standard space estimated using the T1 image is then applied to the EPI data, but this should be studied in future work.

It should also be noted that EPInorm can be applied (optimally) to each functional MRI run. This minimizes concerns about subject motion (and geometric distortion differences) between runs creating increased variability in spatial normalization. Of course, motion within a run would still cause potential issues, and to address this problem, emerging approaches collect distortion correction information more regularly throughout the scan [In et al., 2015; Oh et al., 2012]. Alternatively, one could collect reverse phase encoded images at every other timepoint, however at the cost of cutting the temporal resolution in half. And finally, there are also approaches which jointly estimate multiple factors such as distortion correction and movement interactions or EPI Nyquist ghost effects [Andersson et al., 2017; Xie et al., 2017].

There are several limitations to our approach. We mainly focused on data without the use of distortion correction, primarily because this is the most widespread use of the T1norm approach. In addition, the distortions and signal dropout in the EPI scan can be complex. In some cases, the EPInorm approach may be detrimentally stretching out signal to cover dropout regions that have been lost; in other cases, the EPInorm approach may also be doing a better job of aligning the voxels within the brain. In this empirical study, the “right answer” is relative to the conditions and nature of the data tested. Here, we include several datasets of different types and tasks to increase the generalizability of our conclusions, but some datasets from some scanners may behave differently. We did not exhaustively compare all T1‐based registration algorithms [Klein et al., 2009], including those demonstrably more accurate in published studies such as surface‐based registration [Dale et al., 1999; Fischl et al., 1999; Fischl and Dale, 2000]. However, previous research comparing the impact of T1‐based registration algorithms on prediction and reproducibility metrics derived from group‐level statistical parametric images found that higher order polynomial warps compared to affine alignment had only a minor impact [Strother et al., 2004]. While surface‐based algorithms have been shown to further improve the T1‐to‐T1 warping compared to now standard higher‐order polynomial volume‐based normalization algorithms, they still require proper EPI‐to‐T1 alignment, which is suspect to the warping and signal dropout aspects of the EPI scan, and thus we predict that they would show similar results as the T1norm approach used in this article. However, future studies should more extensively compare the impact of other T1‐based approaches on the analysis of fMRI data. Some newer studies are collecting very high‐resolution EPI images, which may benefit even more from the EPInorm approach; however, if distortions are minimized in such acquisitions then the T1norm approach may provide some benefits. Note, one other advantage of the EPInorm approach is it allowed spatial normalization to proceed without the requirement of the extra T1 scan (which in some cases may not be available).

## CONCLUSIONS

We show results suggesting that the widely used T1norm approach (without distortion correction) does not spatially normalize the EPI data and the EPInorm approach. The differences are striking, consistent across multiple datasets (differing in how the data were collected and processed), and should give pause to those who plan to use the T1norm approach without distortion correction. Interesting, distortion correction substantially improves the results for the T1norm approach, but has much less of an effect on the EPInorm approach, presumably because it is already doing a distortion correction of sorts through the nonlinear transformation.
